# Identification of Ultrasound-Sensitive Prognostic Markers of LAML and Construction of Prognostic Risk Model Based on WGCNA

**DOI:** 10.1155/2023/2353249

**Published:** 2023-02-10

**Authors:** Chuan Tian, Hui Guo, Wei Wei

**Affiliations:** ^1^Department of Ultrasound, The First Affiliated Hospital of Jinzhou Medical University, Jinzhou, China; ^2^Department of Diagnostic Imaging, The First Affiliated Hospital of Jinzhou Medical University, Jinzhou, China; ^3^Department of Hematology, The First Affiliated Hospital of Jinzhou Medical University, Jinzhou, China

## Abstract

**Background:**

Acute myeloid leukemia (LAML) is the most widely known acute leukemia in adults. Chemotherapy is the main treatment method, but eventually many individuals who have achieved remission relapse, the disease will ultimately transform into refractory leukemia. Therefore, for the improvement of the clinical outcome of patients, it is crucial to identify novel prognostic markers.

**Methods:**

The Cancer Genome Atlas (TCGA) and the Gene Expression Omnibus (GEO) databases were utilized to retrieve RNA-Seq information and clinical follow-up details for patients with acute myeloid leukemia, respectively, whereas samples that received or did not receive ultrasound treatment were analyzed using differential expression analysis. For consistent clustering analysis, the ConsensusClusterPlus package was utilized, while by utilizing weighted correlation network analysis (WGCNA), important modules were found and the generation of the coexpression network of hub gene was generated using Cytoscape. CIBERSORT, ESTIMATE, and xCell algorithms of the “IOBR” R package were employed for the calculation of the relative quantity of immune infiltrating cells, whereas the mutation frequency of cells was estimated by means of the “maftools” R package. The pathway enrichment score was calculated using the single sample Gene Set Enrichment Analysis (ssGSEA) algorithm of the “Gene Set Variation Analysis (GSVA)” R package. The IC_50_ value of the drug was predicted by utilizing the “pRRophetic.” The indications linked with prognosis were selected by means of the least absolute shrinkage and selection operator (Lasso) Cox analysis.

**Results:**

Two categories of samples were created as follows: Cluster 1 and Cluster 2 depending on the differential gene consistent clustering of ultrasound treatment. The prognosis of patients in Cluster 2 was better than that in Cluster 1, and a considerable variation was observed in the immune microenvironment of Cluster 1 and Cluster 2. Lasso analysis finally obtained an 8-gene risk model (GASK1A, LPO, LTK, PRRT4, UGT3A2, BLOCK1S1, G6PD, and UNC93B1). The model acted as an independent risk factor for the patients' prognosis, and it showed good robustness in different datasets. Considerable variations were observed in the abundance of immune cell infiltration, genome mutation, pathway enrichment score, and chemotherapeutic drug resistance between the low and high-risk groups in accordance with the risk score (RS). Additionally, model-based RSs in the immunotherapy cohort were significantly different between complete remission (CR) and other response groups.

**Conclusion:**

The prognosis of people with LAML can be predicted using the 8-gene signature.

## 1. Introduction

Acute myeloid leukemia (LAML) is a heterogeneous hematological cancer distinguished by the interruption of myeloid differentiation and the accumulation of mother cells in the bone marrow. Its main feature is that the proliferation of microleukoblasts or leukocytes cannot differentiate normally [1]. LAML, the most prevalent form of acute leukemia in adults, is highly heterogeneous and prone to recurrence [2, 3]. According to the latest data, it is estimated that by 2022, there will be 20050 new cases of LAML and about 11540 mortalities in the United States alone [4]. For decades, the conventional treatment of primary LAML has been to induce chemotherapy first in order to achieve complete remission (CR), and then give consolidation and intensive treatment after remission, or choose stem cell transplantation at a selected time [5]. Among them, about 40–90% of LAML patients respond to the initial induction chemotherapy and can undergo CR [6–9]. However, the remission rate of young patients is only 40%–50% [9]. Chemotherapy resistance is one of the important obstacles for patients with LAML to achieve long-term remission after treatment [10, 11]. Therefore, for a better understanding of the molecular characteristics involved in the occurrence of LAML and for the improvement of a patient's clinical outcome, it is essential to explore new prognostic markers.

The advantages of convenience and small side effects make ultrasound the second most widely used imaging method in the world. In addition to being widely used in diagnostic imaging, ultrasound is also often used in the treatment of various diseases [12]. With the development of ultrasound molecular imaging technology in recent years and its clinical application, this technology can be used to provide more accurate diagnosis and treatment for tumor patients, which is expected to improve the treatment failure caused by chemotherapy resistance. Ultrasound-mediated targeted delivery (UMTD) is a novel therapeutic material delivery approach based on ultrasound that has enormous potential for effective drug delivery and considerably enhancing drug treatment impact [13]. Increasing the distribution and absorption of chemotherapy drugs through the use of ultrasonic contrast agents (UCAs) as carriers is essential for improving the chemotherapy efficacy of tumor patients [12]. Advanced cervical cancer is treated with brachytherapy, and using ultrasonography during brachytherapy significantly improves the prognosis for cervical cancer patients [14]. Additionally, ultrasound combined with micro/nanobubbles can transfer genes and antigens to cells, which may effectively increase the response of tumors to immunotherapy [15]. Simultaneously, studies have confirmed that ultrasound technology can also enhance the therapeutic effect of radiotherapy and photodynamic therapy (PDT) [16, 17]. Automated breast ultrasound (AUBS) and digital breast tomosynthesis (DBT) can be used to assess and track the efficacy of breast cancer patients in terms of prognosis [18]. Ultrasound technology can also be used for the prediction of a cancer patient's response to chemotherapy and to evaluate its correlation with long-term survival before treatment, helping to provide a more accurate diagnosis and treatment for patients [19, 20]. In a word, ultrasound diagnosis and treatment technology will benefit more and more tumor patients. Studying the principle and biological significance of its impact on prognosis and survival in cancer can further play the role of this technology in tumor diagnosis and treatment.

Differential expression analysis was used in this study for the identification of the differential sensitive gene of LAML before and after ultrasound treatment. Two ultrasound-sensitive subtypes with significant prognostic differences were identified based on this gene. Furthermore, an 8-gene risk model including GASK1A, LPO, LTK, PRRT4, UGT3A2, BLOCK1S1, G6PD, and UNC93B1 was constructed for the evaluation of the prognosis of patients with LAML. The model has good and stable prognostic evaluation efficiency.

## 2. Materials and Methods

### 2.1. Data Set Source and Preprocessing

The expression profile data (FPKM value) and clinical data (Table 1) of LAML were accessed from the Cancer Genome Atlas (TCGA) database with the help of the “TCGAbiolinks” R tool. The FPKM value was log2-converted, and the unified survival time unit was used when processing survival information: days.

Both the expression profile and ultrasonic grouping data for GSE10212 and the clinical information for GSE71014 were accessed from the Gene Expression Omnibus (GEO) database (https://www.ncbi.nlm.nih.gov/geo/), and the following processing was followed: (1) the samples with no clinical follow-up information were discarded; (2) the samples with no records of survival time (<0 days) and no survival status either were rejected, while the unified unit of survival time was days; (3) the probe was turned into a gene symbol; (4) the probe that was associated with multiple genes was eliminated; (5) the median value was calculated for the expression having multiple gene symbols.

The “IMvigor210CoreBiologies” R package was employed to download the expression profile and survival and response information of the IMvigor210 immunotherapy cohort (bladder cancer), and samples with survival information and expression data were selected for analysis.  Table 2 illustrates the clinical information.

### 2.2. Differential Expression Analysis

The differential expression analysis in this study was carried out with the help of the “limma” R package. In the GSE10212 dataset, differential expression analysis was performed on samples that received ultrasound treatment and those that did not receive ultrasound treatment, and DEGs were identified with a threshold *p* value <0.01 (because no differential gene met adjust.*p* value <0.05) and |log2FC| > 0.585. For the differential expression analysis of TCGA ultrasound-sensitive subtypes, the Benjamini–Hochberg (FDR) corrected adjust.*p* value <0.01 and |log2FC| > 0.585 were utilized to identify DEGs.

### 2.3. GO and KEGG Enrichment Analysis

The GO and KEGG enrichment analyses were conducted with the help of the “clusterProfiler” R package on the differential genes obtained from the two differential expression analyses. *p* adjust method was set as BH, and an adjust.*p* value <0.05 was used as a cutoff to identify substantially enriched pathways. According to an adjust. *p* value <0.05, the top 10 pathways with significant enrichment were selected for visualization.

### 2.4. Unsupervised Cluster Analysis

Consistent clustering analysis was performed on TCGA-LAML samples by using the “ConsensusClusterPlus” R package to identify ultrasound-sensitive molecular subtypes. The analysis process adopted an 80% resampling rate and 1000 repetitions to assure classification stability. Moreover, the survival curve of KM was drawn by the “survival” R package, and the significance of the prognosis variation between typing was verified by the log-rank test. Finally, the clustering outcomes with a good clustering effect and significant differences in survival among subtypes were selected as the subtype recognition results.

### 2.5. Weighted Gene Coexpression Network Analysis (WGCNA)

The association pattern between gene expression in microarray or RNA-seq is commonly characterized by utilizing WGCNA. The gene coexpression network of complex biological processes was divided by WGCNA into a number of highly associated feature modules, which represent various groups of highly synergistic gene sets. These modules can be associated with specific clinical features for the identification of genes with key functions, helping to study potential mechanisms underlying specific biological processes, and exploring candidate biomarkers.

“WGCNA” R package was used to identify the hypervariable gene of adjust.*p* value <0.01 in the ultrasound-sensitive molecular subtype of the TCGA-LAML cohort. Combined with clinical characteristics (age, sex, and survival status), a gene coexpression network was constructed, and key modules were identified through the correlation coefficient between clinical characteristics and modules. The hub genes of key modules were then identified according to the GS and MM values, and the coexpression network of hub genes was created by using Cytoscape software.

### 2.6. Construction of Prognostic Risk Model and Analysis of Survival Differences

As per the hub gene of the key module, the univariate Cox analysis was conducted to determine (*p* < 0.05) the indicators associated with the prognosis of LAML. Simultaneously, the median expression of a single signature was taken as the threshold point, and two groups of LAML samples were created as follows: high and low expression groups. The KM method was employed to build the prognosis analysis survival curve, and the log-rank test was utilized to calculate the significance of the difference. The Lasso regression method of the “glmnet” R package was employed to identify the important prognosis-related genes, and the prognosis model was created. The tumor samples were divided into two groups as follows: high-risk and low-risk groups using the median RS as the threshold, and the KM method was employed to generate the survival curve of prognosis analysis, whereas the log-rank test was employed to determine the significance of the difference. The receiver operating characteristic (ROC) curve was created using the R package “timeROC” to evaluate the scoring prediction by the disturbance scoring model, while the “ggplot2” R package was utilized to produce the scatter diagram of survival time and survival state, and the scatter diagram of sample score as well. In addition, the “pheatmap” R package was utilized to create the expression heat map of model genes. The expression value of each candidate gene was added together and multiplied by the weight to determine the model's risk value. The formula is as follows: RS=∑_*i*=0_^*n*^coef(*i*) × Exp(*i*).

### 2.7. Estimation of Proportion of Immune Infiltrating Cells and Immune Score

Three algorithms from the “IOBR” R package, CIBERSORT, ESTIMATE, and xCell, were employed to measure the degree of immune infiltrating cells on the basis of the expression profile of the TCGA-LAML dataset [21].

CIBERSORT algorithm is a method used in complex tissues for the characterization of cell composition depending on the expression profiles of genes. The leukocyte characteristic gene matrix LM22 evaluated 547 genes to determine the differentiation between 22 immune cell types, comprising myeloid subpopulations, natural killer (NK) cells, plasma cells, immature and memory B cells, and 7 different types of T cells. The LM22 characteristic matrix and CIBERSORT were used to estimate the proportion of the 22 cell phenotypes in the sample, and when added, the resulting sum of all immune cell types was 1 in each sample.

The ESTIMATE algorithm was employed to measure the immune score, tumor purity, matrix score, and estimate score of the tumor.

XCELL can carry out cell type enrichment analysis on the basis of the gene expression data of 64 different immune cell and stromal cell types. In order to reduce the correlation between closely related cell types, the XCell machine learns from thousands of different cell types from different sources based on gene signature. Through extensive computer simulation of signature and cellular immune typing, xCell can reliably describe the landscape of cellular heterogeneity of tissue expression profile.

### 2.8. Genome Mutation Analysis

“Maftools” R package, combined with clinical grouping information, was used to draw a waterfall diagram to show the variation distribution of genes with high somatic mutation frequency in LAML samples and to classify the samples with model grouping information to draw a waterfall diagram.

### 2.9. HALLMARK Pathway Enrichment Analysis

With the help of the ssGSEA algorithm of the “GSVA” R package, the enrichment score of 50 hallmark pathways for each sample was evaluated in accordance with the gene expression of LAML samples. The correlation of expression and enrichment score of the RS and model genes were determined with the cor function, and the “corrplot” R package was used to visualize the results. Moreover, the statistical tests were employed for calculating the enrichment score differences between the model groups, while the enrichment score heat map was produced by combining the clinical characteristics of the samples with the “pheatmap” R package.

### 2.10. Drug Sensitivity Analysis

Combined with the expression data of model genes, the sensitivity (IC50 value) of 138 drugs in the Genomics of Drug Sensitivity in Cancer (GDSC) dataset was predicted by using the “pRRophetic” R package. The sensitivity of patients with LAML to drug treatment was evaluated by the IC50 value. The Wilcoxon test was employed for comparing the IC50 values between both risk groups, and the drugs that differed substantially between the two groups were identified.

### 2.11. Statistical Test

The Wilcoxon test was utilized for comparing variations between the two groups of samples when marking the significance, and the Kruskal–Wallis test was employed for the comparison of variations between various groups of samples, where ns represents *p* > 0.05, ^*∗*^ represents *p* ≤ 0.05, ^*∗∗*^represents *p* ≤ 0.01, ^*∗∗∗*^represents *p* ≤ 0.001, and ^*∗∗∗∗*^represents *p* ≤ 0.0001. Among them, *p* < 0.05 was significant.

## 3. Results

### 3.1. Identification of Ultrasound-Sensitive Genes in the GEO Dataset

**Figure 1:**
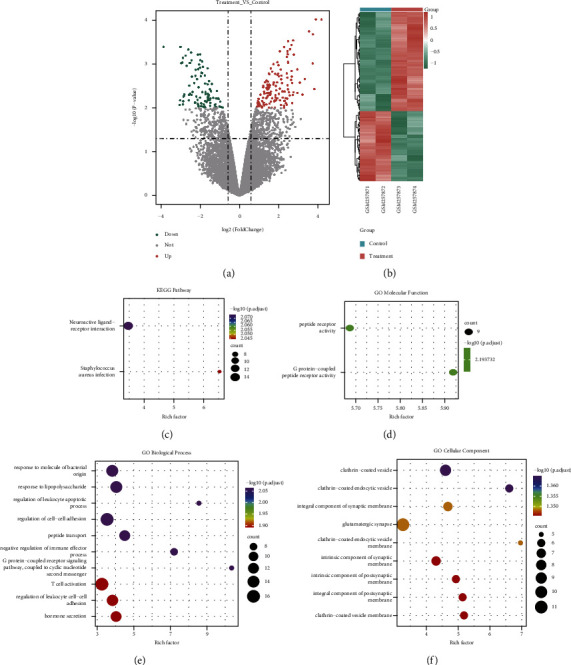
GSE10212 dataset's differential expression and functional enrichment analyses results: (a) volcano map of DEGs in ultrasound and nonultrasound groups; (b) in the heat map of DEGs, high and low expressions are represented by red and green, respectively; (c–f) bubble diagram of enrichment pathway of KEGG pathway, molecular function (MF), biological process (BP), and cell component (CC) of DEGs, in which the number of enriched differential genes is represented by the point's size and the color characterizes the significance of enrichment results.

Differential expression analysis was performed on ultrasound and nonultrasound samples of the GSE10212 dataset to select ultrasound-related differential genes. A total of 227 significantly different genes were obtained, including 133 differentially overexpressed genes and 94 differentially downregulated genes. A volcanic map and heat map were generated to show the expression and distribution of DEGs among subtypes (Figures 1(a) and 1(b)). Moreover, KEGG enrichment analysis and GO function enrichment analysis were carried out on the identified DEGs. The results are shown in Figures 1(c)–1(f), for the analysis results with enrichment entries greater than 10, the TOP10 entries with significant enrichment results were selected to draw a bubble diagram, Fig. C is the enrichment results of the KEGG pathway, and Figures 1(d)–1(f) are the enrichment results of GO's molecular function, biological process, and cell components, respectively. It demonstrated that these genes are enriched in biological processes such as regulation of cell-cell adhesion, T cell activation, negative regulation of immune effector process, and pathways related to neuroactive ligand-receptor interaction and Staphylococcus aureus infection.

### 3.2. Identification of Ultrasound-Sensitive Subtypes in TCGA Cohort

**Figure 2:**
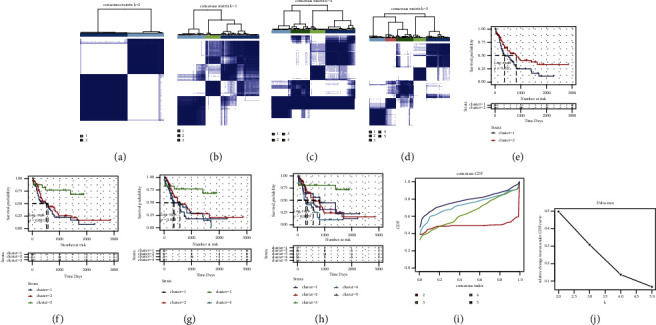
Subtype identification results of TCGA-LAML queue: (a–d) clustering results when the classification number *k* = 2, *k* = 3, *k* = 4, and *k* = 5; (e–h) survival curves among subtypes when classification number *k* = 2, *k* = 3, *k* = 4, and *k* = 5; (i) CDF curve distribution of consistent clustering; (j) the distribution of the area under the CDF curve of consistent clustering.

Ultrasound-sensitive DEGs were detected in the GEO dataset, and the TCGA-LAML cohort was used for the molecular subtypes identification by consistent clustering. The clustering effect was the best when the KM was the clustering algorithm, whereas euclidean was the distance, best K = 2 (Figures 2(a) and 2(d)). The cumulative distribution function (CDF) of consistent clustering is exhibited in Figure 2(i), which shows the cumulative distribution function when *k* took different values.  Figure 2(j) shows the change of the area under the CDF curve when *k* was relative to *k* − 1. Furthermore, two independent ultrasound-sensitive molecular subtypes with significant survival differences were identified, and the prognosis of cluster2 (C2) was substantially better than that of cluster1 (C1) (Figures 2(e)–2(h)).

### 3.3. Differences in the Expression of Ultrasound-Sensitive Molecular Subtypes and Immune Infiltration

**Figure 3:**
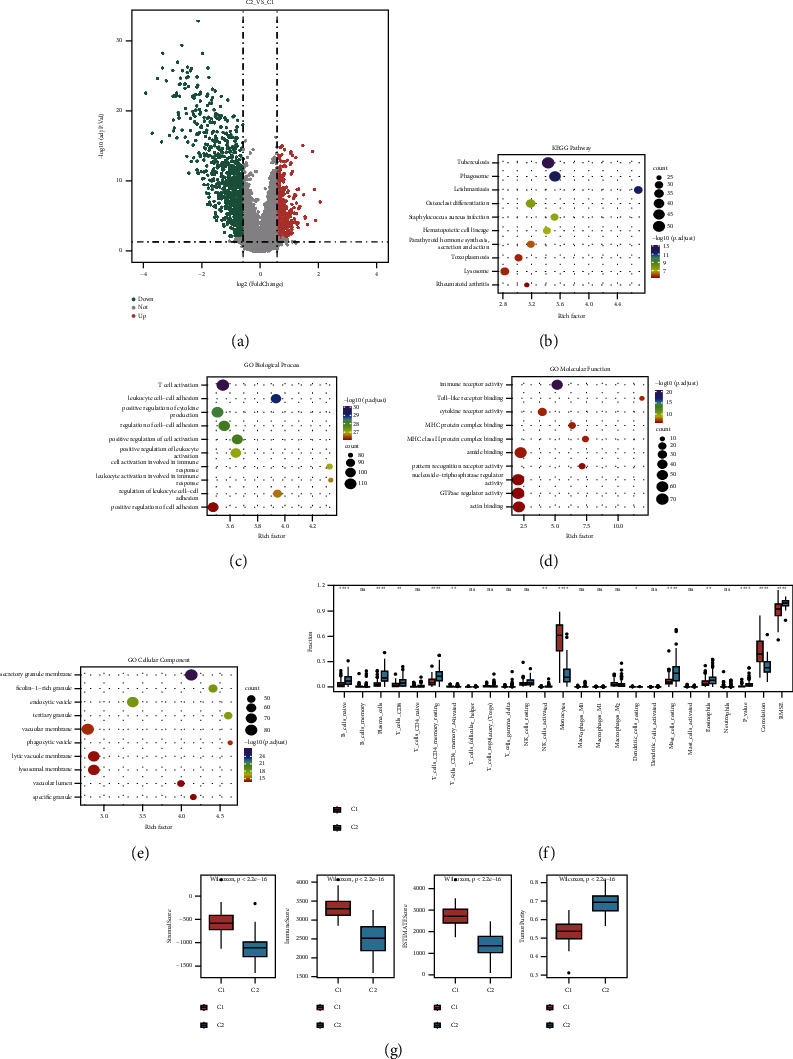
Differential results of expression and immune infiltration of ultrasound-sensitive subtypes: (a) a volcanic map of differential expression among subtypes. Upregulation is represented by red, downregulation is represented by green, and genes with no statistical difference are represented by gray; (b) in the bubble diagram of the KEGG pathway enrichment analysis of DEGs, the number of genes enriched is represented by the size of the dot, and the enrichment significance is represented by the color; (c–e) bubble diagram of GO function enrichment analysis of DEGs, which are BP, MF, and CC; (f) box diagram of immune infiltration difference between ultrasound-sensitive subtypes, red is C1 and green is C2; (g) box diagram of the distribution difference of matrix score, immune score, ESTIMATE score, and tumor purity between ultrasound-sensitive subtypes, red is C1 and green is C2.

The clinical significance of ultrasound-sensitive subtypes was explored by analyzing the expression differences and immune microenvironment differences among subtypes again. Firstly, differential expression analysis was carried out on subtypes to identify DEGs, and a total of 1341 differential expression genes were obtained, including 375 overexpressed genes and 966 downregulated genes. The volcanic map is shown in Figure 3(a). Moreover, DEGs were subjected to KEGG enrichment analysis and GO function enrichment analysis, the top 10 pathways with enrichment significance were selected to draw a bubble diagram. The results are shown in Figures 3(b)–3(e), and they were mainly enriched in items related to immune regulation, such as leukocytes, hematopoietic cells, immune response, and MHC molecules. Subsequently, by determining the degree of immune cell infiltration and the predicted immune-related score, the differences in the tumor immune microenvironment between subtypes were investigated. The findings demonstrate that the CIBERSORT algorithm predicted 22 types of immune cell infiltration ratios, and 13 of those types had substantial variances between subtypes (Figure 3(f)), the box diagram of matrix score, immune score, estimate score, and tumor purity, respectively (Figure 3(g)). The differences between the four scores were statistically significant, with the three C1 scores exceeding C2, while the tumor purity of C1 was lower than C2.

### 3.4. WGCNA in Identifying Key Modules and Hub Genes

**Figure 4:**
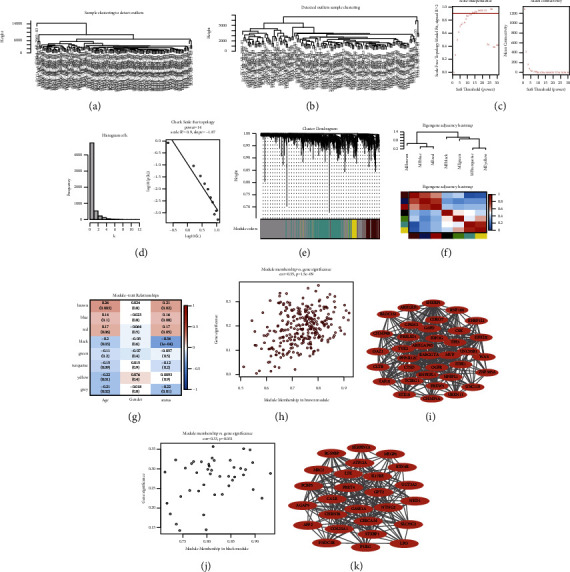
The results diagram of weighted gene coexpression network analysis: (a, b) sample clustering tree before and after removing outliers; (c) soft threshold distribution scatter diagram, the weight is represented by soft threshold (power), and the correlation and average connectivity between connectivity *k* and *p*(*k*) is represented by the ordinate; (d) soft threshold inspection diagram; (e) the clustering tree graph of genes in the module. The upper part of the graph is the clustering tree of genes, and the lower part is the module gathered according to similarity; (f) feature vector gene clustering tree graph and module correlation heat map; (g) module character correlation heat map; (h–k) the scatter diagram of GS and MM value distribution in the module. The row represents the module, the column is the trait, and the value is the correlation coefficient.

The highly mutated genes among ultrasound-sensitive molecular subtypes in TCGA-LAML were selected for WGCNA analysis. 130 LAML samples were clustered (Figure 4(a)), while cut height was set to 8000 to eliminate outliers; finally, 126 samples were used for subsequent analysis.  Figure 4(b) shows the clustering tree after removing outliers. As shown in Figure 4(c), when the correlation coefficient was greater than 0.9, the optimal soft threshold was 14. Additionally, Figure 3(d) shows that K has a negative correlation with *p* (*k*) (correlation coefficient: 0.9), which indicates that a gene scale-free network can be established by the selected *β* value. Furthermore, in the module, the minimum gene number was set to 30, the maximum distance of the module to 0.25, and the calculation methods of coexpression correlation and module trait correlation were Pearson.  Figure 4(e) shows the module clustering tree and it can be observed that brown is a more important module. The feature vector gene clustering tree and heat map were drawn, and their results in Figure 4(f) reveal the modules with correlation coefficient >0.8 (dissimilarity coefficient <0.2) would be merged in the subsequent analysis.  Figure 4(g) is the heat map of the correlation between modules and traits, and it can be observed that the key traits are age and status, and the key modules are brown and black. A scatter diagram was drawn to show the linear relationship between GS and MM in the module, and the results are illustrated in Figures 4(h) and 4(j), revealing the correlation coefficients of 0.35 and 0.33, respectively. According to the distribution of GS and MM values of genes in the module, the threshold was set GS > 0.2&MM > 0.8, and hub genes were selected according to the key modules of each key trait. Furthermore, 37 hub genes were selected from the age-brown module and 26 hub genes were selected in the status-black module, and based on the edge file and node file obtained from the export network to Cytoscape function in WGCNA, the hub genes were screened and introduced into Cytoscape to construct the module hub genes coexpression network diagram of a key trait (Figures 4(i) and 4(k)).

### 3.5. Construction and Verification of the Ultrasound-Related Prognostic Signature Recognition of Prognostic Signature Based on Hub Genes

**Figure 5:**
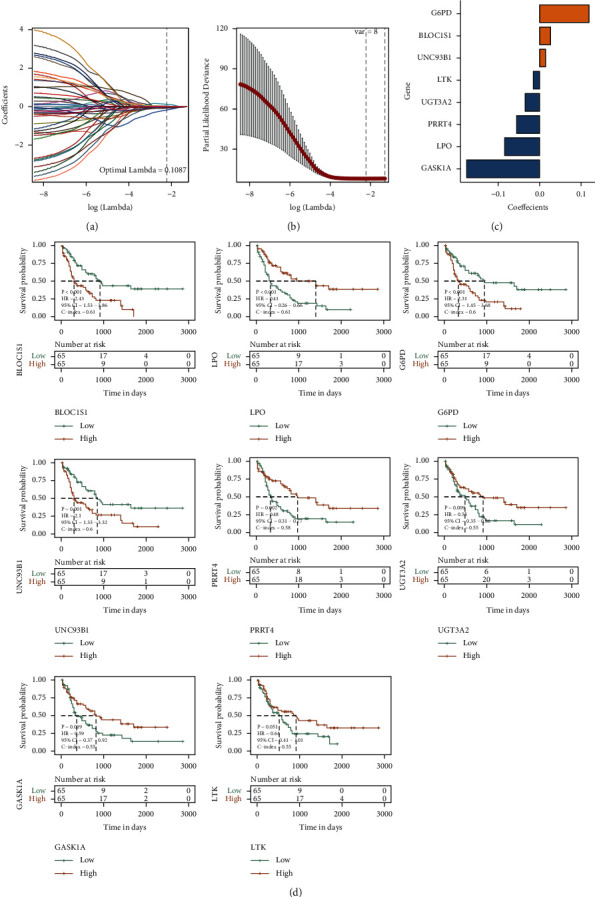
Results of TCGA univariate Cox analysis and Lasso regression analysis: (a) the change track of the Lasso regression independent variable, the logarithm of the independent variable Lambda is represented by the abscissa, and the coefficient of the independent variable is represented by the ordinate; (b) the confidence interval under each Lambda in Lasso regression; (c) Lasso regression coefficient of key prognostic genes; (d) KM curve of the prognostic signature obtained by Lasso regression analysis.

In TCGA-LAML, identification of 63 hub genes was done using univariate Cox analysis, while the threshold *p* < 0.01 was set. Moreover, 45 prognosis-related genes were obtained as well. The median expression of each gene was used as the cutoff value for high and low groups, and the KM survival curve was drawn. Subsequently, through random sampling, 7/10 of the TCGA-LAML overall set (*n* = 130) was selected as the training set (*n* = 91), and on the basis of these 45 prognostic-related signatures, the seed was set at 12110, while Lasso linear regression method was used to remove redundant genes and build a risk model. The results are shown in Figures 5(a)–5(c). Conclusively, 8 prognostic signatures were selected.  Figure 5(d) shows the KM survival curves of 8 prognostic signatures.

### 3.6. Verification of Robustness of Risk Model by Internal Verification Set

**Figure 6:**
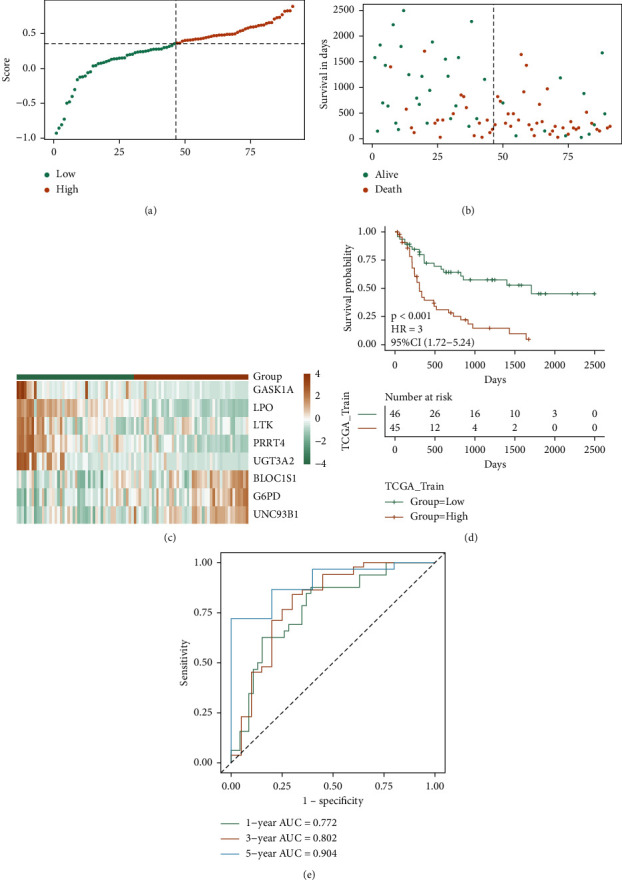
TCGA training set verifying the prognostic efficacy of the model. (a–c) the risk triple plot of the TCGA training set, which is the risk dispersion plot, the survival time scatter plot, and the expression heat map of model genes in the RS group, respectively. Yellow is the high-risk group, while green is the low-risk group; (d-e) KM curve (yellow for the high-risk group and green for the low-risk group) and ROC curve of TCGA training set.

Further assessment of the model scores' impact constructed by the eight signatures on the training set's OS, the median of RS was taken as the critical value, and the samples were distributed into two groups: high-risk and low-risk groups. The scatter plots of survival time and survival state of the training set and the scatter plot of samples' RS were then drawn, respectively. Combined with these two scatter plots, the relationship between survival and score can be observed (Figures 6(a) and 6(b)), whereas the model gene expression of the training set is variable in both risk groups (Figure 6(c)). Moreover, the model's prognostic efficiency was checked by the construction of KM and ROC curves (Figures 6(d) and 6(e)). The prognosis of the samples in the high-risk group was worse, and the *p* < 0.01 of the KM curve of both groups indicates that there is a considerable variation in the prognosis of the two groups. The risk model-based AUC values for the 1-, 3-, and 5-years periods were 0.772, 0.802, and 0.904, respectively, indicating that the model score's prediction efficiency is excellent.

**Figure 7:**
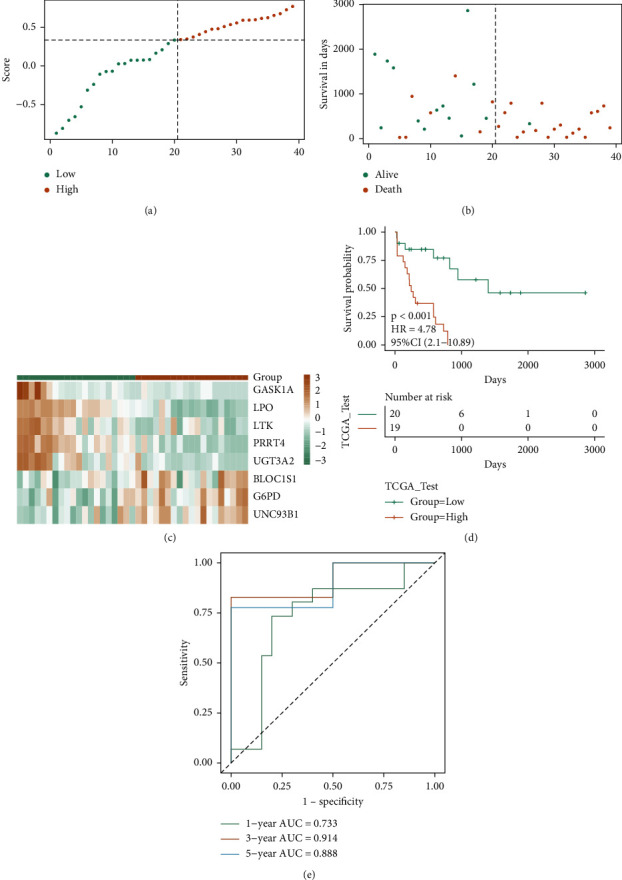
TCGA test set verifying the prognostic efficacy of the model: (a–c) the risk triple plot of the TCGA test set, which is the risk dispersion plot, the survival time scatter plot, and the expression heat map of model genes in the RS group, respectively. The yellow color is for the high-risk group and the green color for the low-risk group; (d-e) KM curve (yellow for the high-risk group and green for the low-risk group) and ROC curve of the TCGA test set.

Additionally, the test set of TCGA-LAML was employed to check the ability of RS for OS prediction. In accordance with the same method as the TCGA training set, two sample groups were created as follows: the high-risk group and the low-risk group, and the survival differences were compared. The scatter diagram of survival time and survival state of the test set, the scatter diagram of sample RS, and the heat map of model gene expression were studied in both risk groups of the test set (Figures 7(a) and 7(c)). The high-risk group's prognosis was observed to be worse than that of the low-risk group, and substantial differences were observed in the prognosis of both groups (Figures 7(d) and 7(e)). In the TCGA test set, the respective AUC values of 1-, 3-, and 5-years were 0.733, 0.914, and 0.888, respectively. These results confirm that the prognostic efficacy of the TCGA test set model is stable and good.

**Figure 8:**
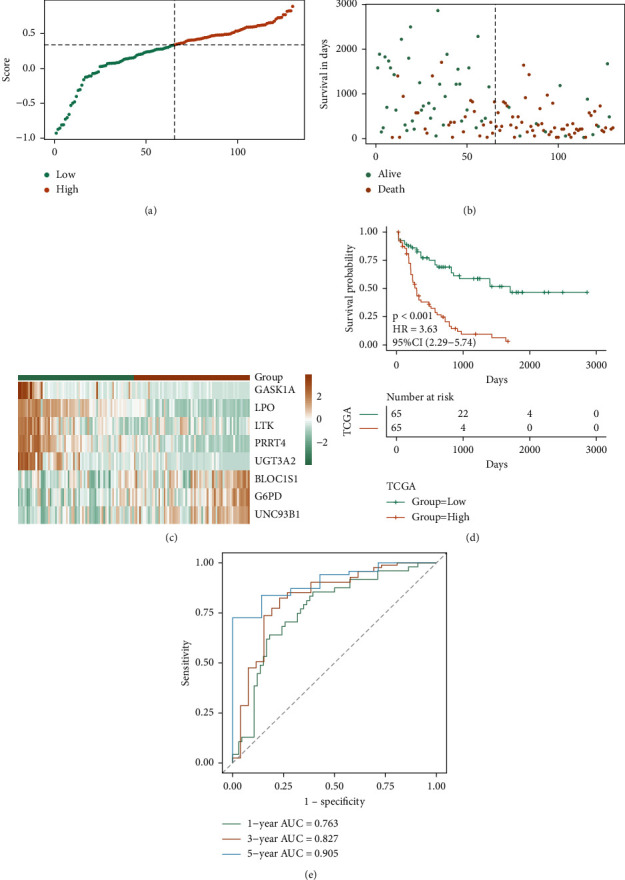
TCGA whole set verifying the prognostic efficacy of the model. (a–c) the risk triple plot of TCGA whole set, which is the risk dispersion plot, the survival time scatter plot, and the expression heat map of model genes in the RS group, respectively. The yellow color is for the high-risk group, while the green color is for the low-risk group; (d-e) KM curve (yellow for the high-risk group and green for the low-risk group) and ROC curve of TCGA whole set.

Finally, the whole set of TCGA-LAML was employed to check the ability of RS for OS prediction. Similarly, based on the same method of TCGA training set, two groups of samples were created as follows: the high-risk group and the low-risk group in the overall set. Moreover, the scatter diagram of the survival time and survival state of the whole set, the scatter diagram of the RS sample, and the heat map of the expression of model genes were studied in both risk groups of the whole set (Figures 8(a)–8(c)). Additionally, it was observed that the prognosis of the high-risk group is worse, and considerable variation was observed in the prognosis of the two risk groups (Figures 8(d) and 8(e)). In the overall concentration of TCGA, the AUC of 1-, 3-, and 5-years was 0.763, 0.827, and 0.905, respectively. The above results confirm that the prognosis of the TCGA integrated model is stable and good.

### 3.7. The External Validation Set Verifying the Prognostic Efficacy of the Model

**Figure 9:**
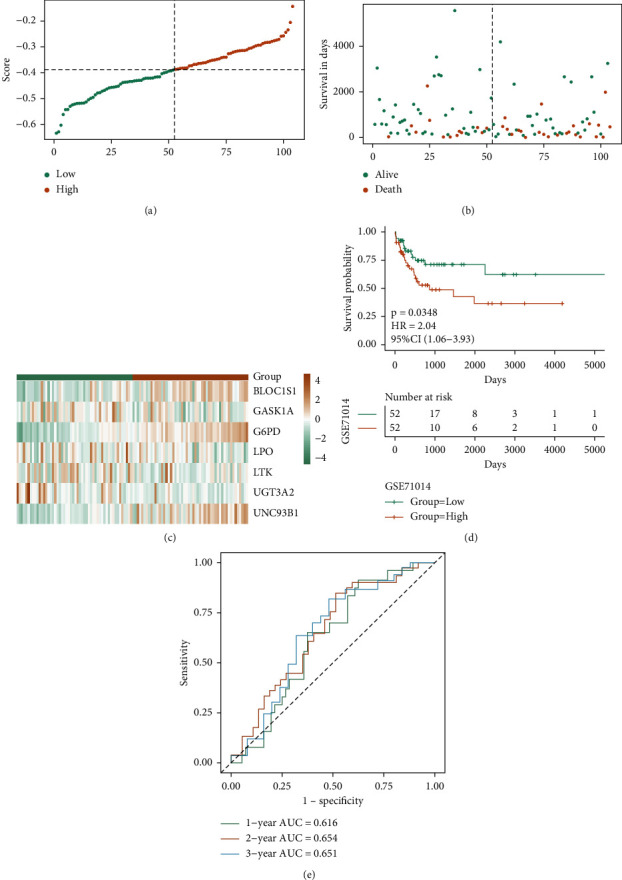
GEO dataset verifying the prognostic efficacy of the model: (a-c) the risk triple plot of the GSE71014 dataset, which is the risk dispersion plot, the survival time scatter plot, and the expression heat map of model genes in the RS group, respectively. Yellow color for the high-risk group, while green for the low-risk group; (d-e) KM curve (yellow for the high-risk group and green for the low-risk group) and ROC curve of the GSE71014 dataset.

The robustness of the model score can be further verified by the prediction of the OS of patients with LAML. This study selected a GEO external data set for the same analysis and verification. Figures 9(a) and 9(b) illustrate the results of the scatter plot of survival time and survival state and the scatter plot of sample risk score. Combined with these two scatter plots, the relationship between survival and score can be observed. Fig. C illustrates the genes' expression model in the GSE71014 dataset in both risk groups.  Figure 9(d) shows the KM curve of GSE71014. The samples in the high-risk group had a worse prognosis, while the KM curve of the two risk groups (*p* < 0.05) indicates substantial variations in the prognosis of both groups. In Fig. E, the AUC values of 1-, 2-, and 3-years are 0.616, 0.654, and 0.651, respectively, indicating that the prognostic efficiency of the model score is good.

### 3.8. Prognostic Risk Models Associated with Multiple Tumor Characteristics RS, an Independent Prognostic Factor

**Figure 10:**
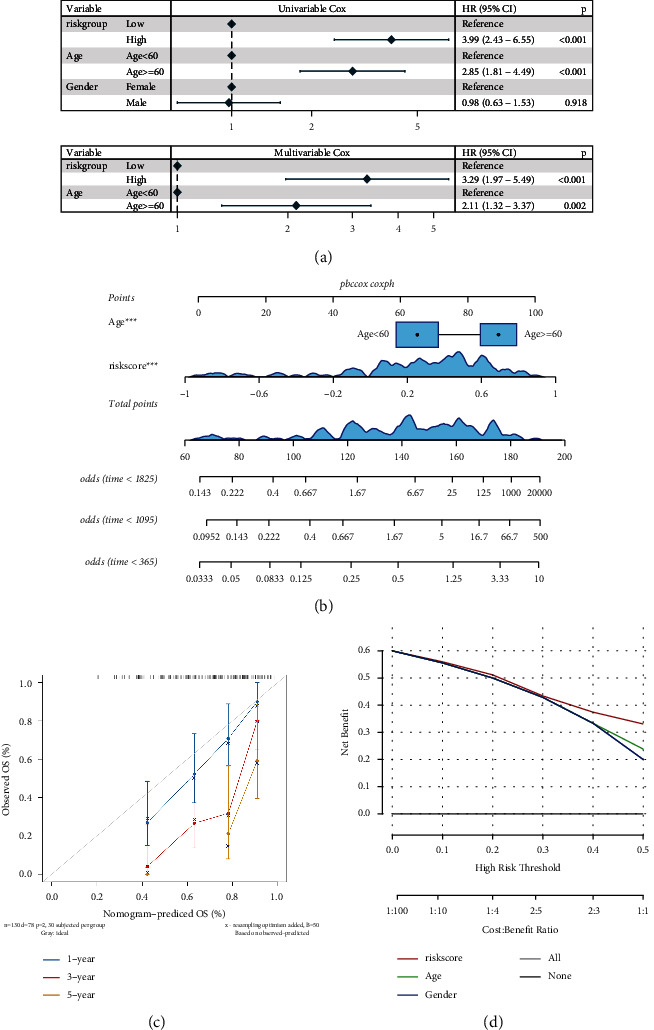
Independence of model scores in clinical characteristics: (a) forest map of the results of univariate and multivariate Cox analysis of clinical factors in TCGA cohort; (b) in the nomogram of the prediction model, the box plus line segment represents the contribution of the clinical factor to the outcome event, total points represent the total score of the sum of the corresponding individual scores after the value of all variables, and the bottom three lines represent the 1-, 3-, and 5-year survival probability corresponding to each value point; (c) calibration curve, the abscissa is the predicted probability and the ordinate is the actual probability. The closer the gray line in the middle is, the more accurate the predicted risk probability is. The lower the gray line is, the lower the risk is underestimated, and the upper part is, the higher the risk is overestimated; (d) the abscissa of the DCA decision curve is the risk probability, and the ordinate is the benefit rate. The curves with different colors represent different clinical factors.

The constructed risk model in this analysis shows good prognostic efficacy in the TCGA dataset and GEO external validation set. Additionally, for verification of RS to be served as an independent prognostic factor, the age and gender of LAML were combined to conduct univariate and multivariate Cox regression analyses. The univariate Cox analysis was performed first, followed by the selection of independent prognostic factors for multivariate Cox analysis. The univariate Cox regression revealed significant variations between the prognostic model group and age group compared with the reference, which proves that they are independent prognostic factors (Figure 10(a)). Furthermore, based on survival time and survival status, nomograms (Figure 10(b)) were constructed in combination with clinical indicators. Age and RS were clinical factors that contributed significantly. The construction of the calibration curve was performed (Figure 10(c)) to assess the nomogram's accuracy. The calibration curve revealed that the prediction accuracy of the model in the 1st and 3rd years is high. DCA decision curves of different classification features were constructed to assess the prognosis accuracy of multiple clinical features. The results are illustrated in Figure 10(d).

### 3.9. The Model Risk Score Related to the Clinical Characteristics of the Tumor

**Figure 11:**
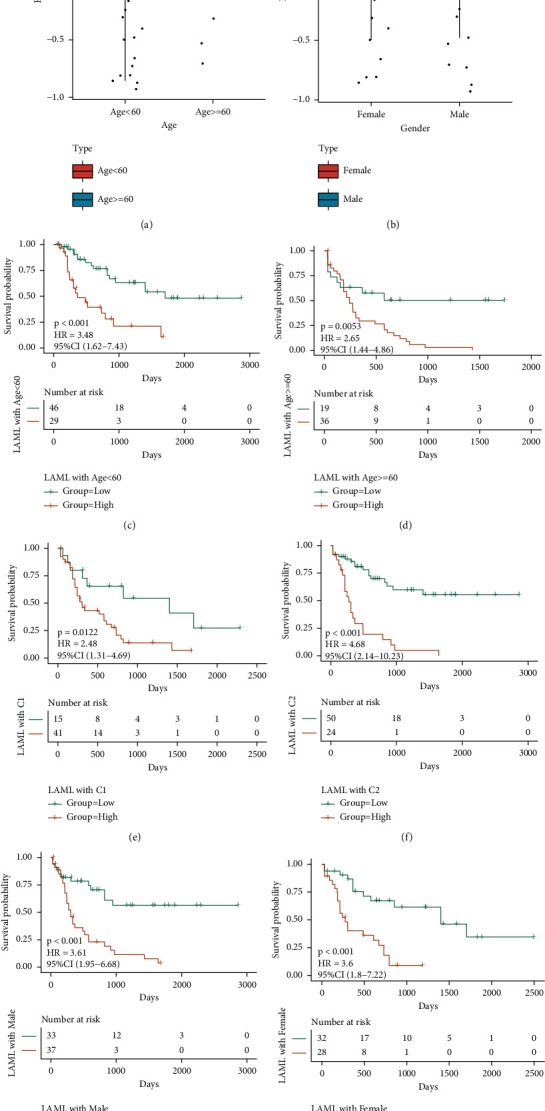
Clinical characteristics related to model scores: (a-b) the distribution of RS in age and gender groups, respectively; (c–h) KM curves of age, cluster, and gender subdatasets in feature grouping, respectively. The yellow color is for the high-risk group and the green is for the low-risk group.

Based on the clinical features of age and gender of the TCGA-LAML dataset, the distribution differences of RS among different clinical feature groups are shown. As shown in Figures 11(a) and 11(b), there are substantial variations in RS in the age group. In addition, according to the grouping information of age, cluster, and gender, the TCGA dataset was divided into two subdatasets, and the KM curves of the subdatasets were drawn, respectively, in accordance with the median value of RS. The KM curves revealed substantial variations in each of the subdataset, and the prognosis of the high-risk group was observed to be worse (Figures 11(c)–11(h)).

### 3.10. The Risk Model Related to the Expression of Immune Checkpoints

**Figure 12:**
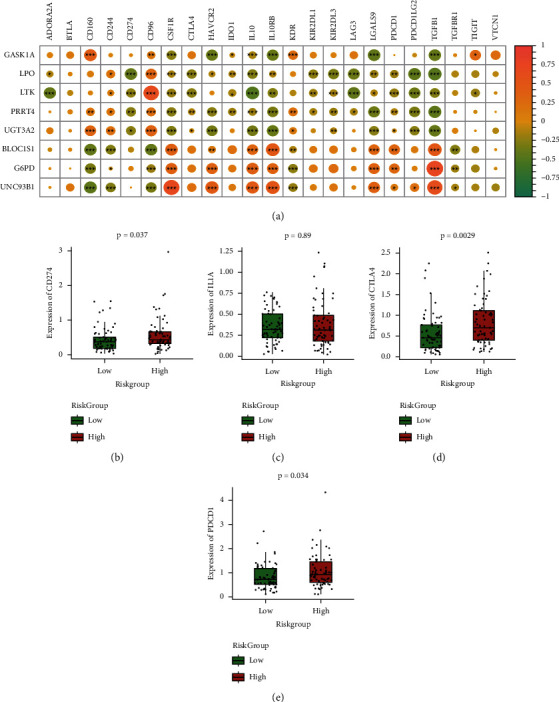
Correlation analysis between model and immune checkpoint: (a) the heat map of the correlation coefficient between the expression of model genes and immune checkpoints (immunosuppressant), the color of the dots represents the correlation, and ^*∗*^represents the significance; (b–e) box diagram of expression differences of four common immune checkpoints between high-risk and low-risk groups. The red color is for the high-risk group and the green color is for the low-risk group.

A group of molecules known as immune checkpoints are expressed in immune cells and have the ability to regulate the level of immune activation while playing a significant role in the occurrence of human autoimmunity as well. The correlation between five types of immune checkpoints was analyzed (from TISIDB, respectively: chemokine, Immunoinhibitor, Immunostimulator, MHC, and receptor) and the expression of eight model genes, and the correlation heat map was constructed as well. The Immunoinhibitor gene is the most commonly used immune checkpoint, and its correlation with model gene expression is shown in Figure 12(a), the model genes generally have a strong correlation with the expression of immune checkpoints. In addition, the box diagram of four common immune checkpoints was drawn, and the variations in the expression of immune checkpoints in model groups were shown through statistical verification. As shown in Figures 12(b)–12(e), there are considerable variations in the gene expression levels of CD274, BTLA, and CTLA4, and the expression level of genes is increased in the high-risk group.

### 3.11. Association between Model Grouping and the Proportion of Immune Infiltrating Cells

**Figure 13:**
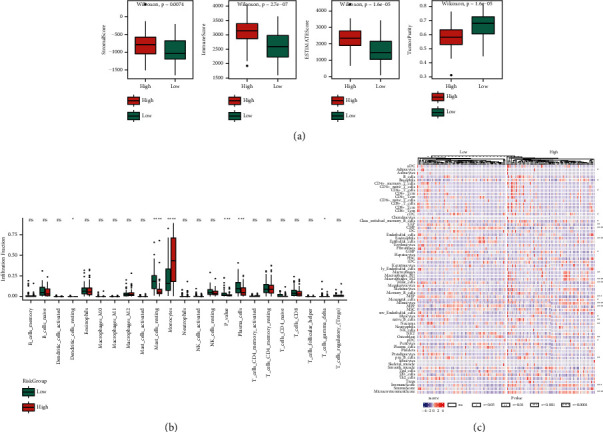
Variation in the proportion of immune infiltrating cells between model groups: (a) box diagrams of matrix score, immune score, ESTIMATE score, and tumor purity of high-risk and low-risk groups, respectively. The red color is for the high-risk group while the green color is for the low-risk group; (b): in the CIBERSORT algorithm, the proportion of immune infiltrating cells in high-risk and low-risk groups is shown in the box diagram. The red color is for the high-risk group while the green color is for the low-risk group; (c) heat map of the difference between high and low-risk groups in the proportion of immune infiltration in the xCell algorithm.

The two primary categories of nontumor constituents in the tumor microenvironment are immune and stromal cells, and they both have the potential to be extremely helpful for tumor diagnosis and prognosis evaluation. Three algorithms were used for calculating the proportion of immune infiltrating cells: immune score, matrix score, tumor purity, and ESTIMATE score. While the tumor purity was reduced in the high-risk group, the findings of the three scores in the high-risk group were noticeably greater in comparison to the low-risk group (Figure 13(a)). Simultaneously, the difference in the proportion of immune cell infiltration in high-risk and low-risk groups was measured using CIBERSORT and xCell algorithms. The immune infiltration difference results of the CIBERSORT algorithm (Figure 13(b)), in which substantial variations were observed in the proportion of immune infiltration of 6 cell types in the high-risk and low-risk groups, whereas the heat map of immune infiltration proportion was constructed based on xCell algorithm (Figure 13(c)), and the infiltration proportion of 24 cell types is considerably variable in high-risk and low-risk groups.

### 3.12. The Expression of Model Genes is Linked with the Proportion of Immune Cell Infiltration

**Figure 14:**
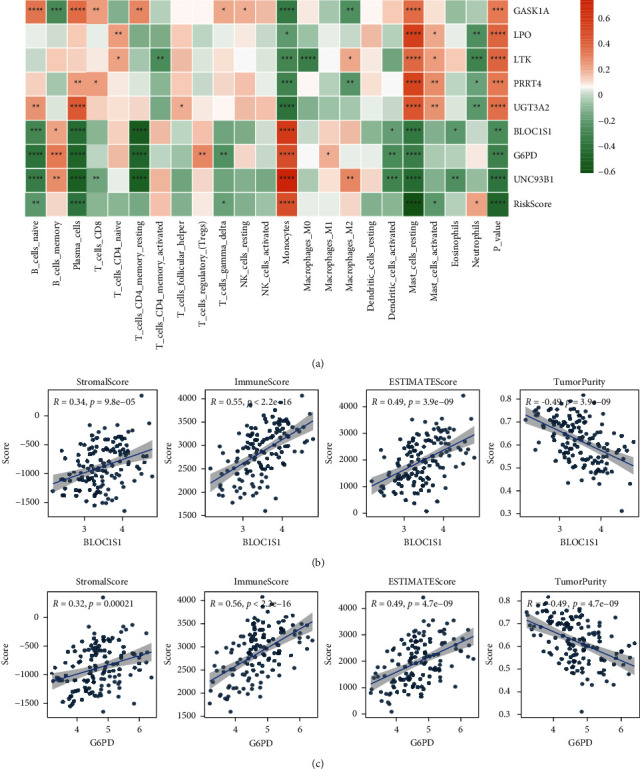
Association between the expression of model genes and the proportion of immune cell infiltration. (a) Heat map of the correlation between gene expression and the proportion of immune infiltrating cells in the model and (b, c) scatter plot of the correlation coefficient between the expression of model genes block1s1 and G6PD and the proportion of immune cell infiltration.

The grouping of risk models depends on the expression of model genes. We can explore the prognosis of cancer affected by the expression of genes by studying the association between the immune microenvironment and model gene expression. According to the proportional analysis of immune cell infiltration by the CIBERSORT algorithm, the significance of gene expression in clinical immunology is represented by calculating the correlation coefficient between the expression of model genes in LAML samples and the proportion of immune cell infiltration. Between the 8 model genes and the proportion of 23 immune cell infiltration, the correlation was illustrated as a heat map (Figure 14(a)). Additionally, a scatter diagram showing the relationship between the ESTIMATE score and the expression of model genes was created, and two model genes with high correlation coefficients were selected for display (Figures 14(b) and14(c)). See the annex for other results.

### 3.13. Differences in Genomic Mutations

**Figure 15:**
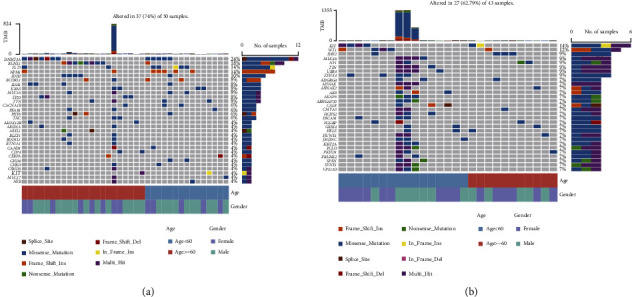
Genome mutation differences among model groups. (a) SNV waterfall plot of TOP30 (mutation frequency) gene in the high-risk group and (b) SNV waterfall plot of TOP30 (mutation frequency) gene in the low-risk group.

Gene mutations can promote and lead to the occurrence of cancer or coordinate to drive the malignant value-added of cancer. The investigation of genome-level mutations is crucial for the development of novel tumor therapies and tumor-targeted drugs. In order to show the distribution of somatic variation among samples between high-risk and low-risk groups and the gene mutation distribution among samples with different clinical characteristics, the TOP30 genes with the highest mutation frequency in the high-risk and low-risk groups were selected to draw a waterfall diagram (Figures 15(a) and 15(b)): the frequency of gene mutation was observed to be substantially higher in the high-risk group than that in the low-risk group.

### 3.14. Model Scores Correlated with Hallmark Pathway Enrichment

**Figure 16:**
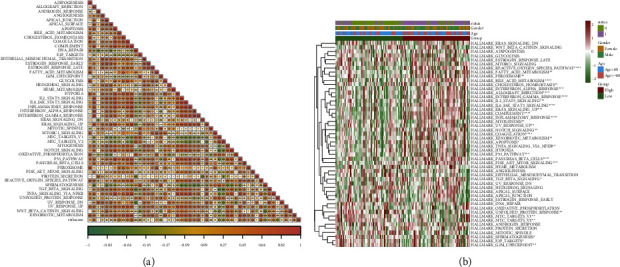
Hallmark pathway enrichment analysis results: (a) correlation heat map of RS and hallmark pathway enrichment analysis, a positive correlation is represented by red, a negative correlation is represented by blue, depth represents high and low correlation, and ^*∗*^represents significance; (b) the enrichment score heat map of Hallmark pathway, ^*∗*^represents the significant difference in the enrichment score of this pathway in the high-risk and low-risk groups.

Based on the expression profile of LAML samples, the hallmark pathway enrichment score results were calculated. Combined with the model score information, the correlation between RS and enrichment score was explored, and the pathway enrichment variation between high and low-risk groups, which is helpful to analyze the association between cancer characteristic pathways and prognosis. The RS was observed to have a significantly positive correlation with the hallmark pathway score (Figure 16(a)), whereas the enrichment scores of 30 pathways (Figure 16(b)) have significant differences among model groups.

### 3.15. Model Score Predicting the Therapeutic Effect of Patients Analysis of Chemotherapeutic Drug Resistance

**Figure 17:**
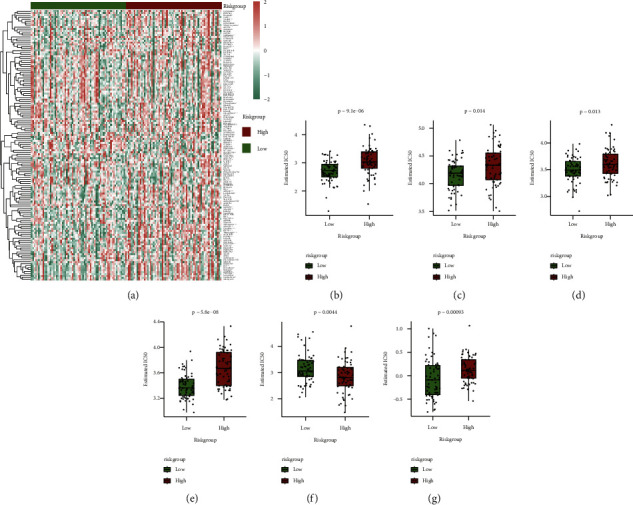
Drug sensitivity differences between model groups: (a) IC_50_ heat map between high and low-risk groups in TCGA-LAML cohort, high drug sensitivity is represented by red, and low sensitivity is represented by green; (b–g): the distribution difference of IC_50_ values of six chemotherapeutic drugs between high-risk and low-risk groups. The red color is for the high-risk group while the green color is for the low-risk group.

The expression profile data of TCGA-LAML was used to predict the sensitivity IC_50_ values of 138 drugs in the GDSC database. A significant difference in IC_50_ values of 60 drugs between high-risk and low-risk groups was observed (Figure 17(a)). According to the model grouping results and IC_50_ values, a box diagram was drawn to show the distribution variations of IC_50_ of drugs between high-risk and low-risk groups, and 6 drugs with considerable variations were selected for display. The results revealed that the IC50 values of high-risk groups are generally higher than those of low-risk groups (Figures 17(b)–17(g)), whereas the results of other drugs are illustrated in the annex.

## 4. Discussion

Acute myeloid leukemia (LAML) is a rapidly developing malignant tumor of the hematopoietic system. It is believed to originate from a single hematopoietic stem cell or progenitor cell. After the normal differentiation process is blocked due to various reasons, it still grows rapidly and divides continuously. These cells are immature and lack normal function, thus affecting the hematopoietic function of the body [22]. Chemotherapy is a crucial therapy for the treatment of tumors, and the main cause of its failure is the development of resistance in tumor cells to chemotherapy [23, 24]. The drug resistance of LAML patients to chemotherapy often manifests in relapse after remission and transformation into refractory leukemia [25]. At present, the molecular mechanism that mediates the transformation of LAML cells from chemotherapy sensitivity to drug resistance is still not completely clear. Therefore, finding biomarkers with prognostic values for LAML is very important for determining the relevant drug targets of treatment intervention and overcoming treatment resistance.

Ultrasound examination can quickly and accurately assess the size and depth of tumors and clarify the extent of involvement of deep tissues [26]. Recently, ultrasonic medicine has broken through the limitations of traditional ultrasonic imaging diagnosis and entered the “nano” era [27, 28]. The deep drug delivery of tumors is also given a new direction by ultrasound-assisted tumor diagnostics and treatment, enhancing local drug concentration to achieve targeted therapeutic goals, and minimizing side effects [29]. Compared with the commonly used response evaluation criteria in solid tumors (RECIST), ultrasound can evaluate the efficacy of antiangiogenesis drugs in tumor patients earlier and more conveniently [30]. In conclusion, ultrasound plays a significant role in the diagnosis, treatment, and prognosis of tumors.

In this study, two ultrasound-sensitive molecular subtypes with significant survival differences were identified based on the differential genes of ultrasonic treatment of LAML, and the prognosis of patients with cluster2 was significantly better than that of cluster1. Then, the immune cell infiltration between different subtypes was further analyzed. The results revealed substantial variations in the immune microenvironment between the two subtypes, which may be the reason for the survival differences between the two subtypes. Then, WGCNA analysis of the disturbed genes between subtypes identified two key modules of the two main clinical features associated with LAML was performed. Finally, based on the prognostic factors significantly related to LAML, we constructed an 8-gene signature RS model composed of GASK1A, LPO, LTK, PRRT4, UGT3A2, BLOCK1S1, G6PD, and UNC93B1 to evaluate the prognosis of patients with LAML. As a secretory protein kinase, GASK1A is expressed in basal epithelial cells, which is not only related to the occurrence and development of tumors but also may cause chemotherapy resistance in some tumor patients [31, 32]. Since polyunsaturated fatty acids and oxygen free radicals react in the body to form lipid peroxide (LPO), and its expression level is correlated with the poor prognosis and disease invasiveness in breast cancer patients [33], studies have revealed that inducing the outbreak of LPO and ferroptosis in tumors can induce the death of drug-resistant cancer cells and effectively improve the efficacy and prognosis of chemotherapy-resistant patients [34]. CLIP1-LTK fusion gene can be used as a therapeutic target of loratinib in patients with non-small cell lung cancer [35]. Abnormal activation and mutation of LTK regulate the growth and apoptosis of tumor cells and affect the occurrence and progression of many types of tumors [36]. LTK is a common up-regulated target gene in stages I-IV of hepatocellular carcinoma, which is mainly involved in tumor immunity and signal transduction [37]. LTK mutations may cause myeloma and can be used as biomarkers to detect specific targets of myeloma [38]. In addition, LTK is closely related to the pathogenesis of LAML [39]. PRRT4 is considered a new prognostic biomarker for gastric cancer [40]. UGT3A2 may be the antidote to polycyclic aromatic hydrocarbons in the human body, and its mutation increases the carcinogenic risk of polycyclic aromatic hydrocarbons [41, 42]. The expression level of UGT3A2 is related to DNA methylation and affects the occurrence and development of LAML [43]. An 11-gene signature, including UGT3A2, established based on the immune microenvironment can be employed for the prediction of the prognosis of thymoma patients [44]. BLOCK1S1, also known as GCN5L1, is a new molecule homologous to the sequence of nuclear acetyltransferase GCN5, which is involved in the regulation of mitochondrial autophagy, fatty acid oxidation, and other mitochondrial biological processes [45–47]. BLOCK1S1 can also regulate the occurrence and development of hepatocellular carcinoma through glutamine metabolism, and the expression level is related to the prognosis of patients [48]. G6PD plays a role in cell cycle regulation (cell growth and death) and is related to tumorigenesis and malignant progression; in addition, it is an indicator of poor tumor prognosis [49, 50]. According to various studies, G6PD can promote the proliferation of LAML cells and patient resistance [51]. Furthermore, the role and mechanism of UNC93B1 in tumors are still unknown and there is no substantial study available. In conclusion, nearly every one of the eight genes examined in this study is strongly linked to the development, progression, and prognosis of different cancers, with LTK, UGT3A2, and G6PD, particularly thought to be crucial in LAML.

This study is the first to develop a prognosis model for LAML based on the differential genes of subtypes that are sensitive to ultrasound therapy, which offers a fresh perspective on the disease's molecular mechanism and prognosis prediction. The model we established is obtained through the comprehensive analysis of multiple datasets, which has high reliability, and the multigene aggregation model has a higher prognostic value than a single gene. However, there are still some limitations to this study. Firstly, the sources of clinical information obtained in this study are TCGA and GEO databases, most of which are white, African, or Latin American, thus, when applying our findings to patients of other races care must be taken. Secondly, because this is a retrospective study, there is no way to avoid some data loss and selection bias. Finally, the model is still in the theoretical stage, and more experiments are needed in the future to further verify the clinical prognostic value of the model.

## 5. Conclusions

In this study, the ultrasound-sensitive subtype of TCGA-LAML was identified based on the ultrasound-sensitive gene for the first time, and finally, an RS model composed of 8 signatures was constructed to evaluate the prognosis of patients with LAML.

## Figures and Tables

**Table 1:** Clinical information table of TCGA LAML queue.

Clinical features	Grouping information	Number of samples
Age	Age ≥60	55
	Age <60	75

Gender	Female	60
	Male	70

Status	Dead	78
	Alive	52

**Table 2:** Clinical information table of IMvigor210 queue.

Clinical features	Grouping information	Number of samples
Status	Dead	189
	Alive	109

Response	CR	25
	PR	43
	SD	63
	PD	167

## Data Availability

The data used to support the findings of this study are available from the corresponding author on reasonable request.
